# The epigenetic effects of breast milk and the association of its nutritional content with maternal diet. Implications for midwifery practice

**DOI:** 10.18332/ejm/97552

**Published:** 2018-10-12

**Authors:** Angeliki Antonakou

**Affiliations:** 1 Department of Midwifery, Midwifery School, ‘Alexander’ Technological Educational Institute of Thessaloniki, Greece

**Keywords:** breastfeeding, epigenetics, maternal diet, infant health

Breast milk has been well proven to be the optimal food for infants up to the first six months of age with the World Health Organisation promoting long-term breastfeeding for the better health and cognitive development of newborns^1^. Breast milk has unique nutritional and nonnutritional benefits that can be partially explained by ‘epigenetics’ although the majority of the underlying biological mechanisms are still unclear^2^. The term ‘epigenetics’ is defined as the influence of environmental factors such as maternal and neonatal nutrition that can lead to heritable changes in the gene expression and therefore to a new phenotypic trait without however modifying the DNA sequence of the individual. The epigenetic effect has been quoted to modulate the individual adaptation to the environment and to influence the lifelong health by modifying inflammatory molecular pathways and the immune response. The main mechanisms of the epigenetic modifications that have been implicated involve the process of DNA methylation, histone modification, and the effect of non-coding RNA^2^.

The link between early nutrition and the epigenetic effects on later adult life has been described in the literature^2,3^. It has been reported that the first two years of life is the time period when epigenetic DNA imprinting activity is considered to be the most active^3^. Early nutrition epigenetic effect has been reported to potentially play a key role in developmental programming and to possibly influence the individual’s susceptibility to the later development of cardiovascular disease, obesity, diabetes, and other chronic conditions^2^. Recent evidence has shown that some of the epigenetic changes ensuing from early nutrition can be transgenerationally inherited, thus having a significant impact on the process of evolution^2^.

There are several reports on the epigenetic effects of the contents of breast milk on the health of the newborns. It has been found that oligosaccharides contained in human milk promote a healthier composition of the infant’s gut microbiota, and therefore play a leading role in programming the infant’s immune phenotype and as a result preventing diseases in early and later life^4^. It has also been reported that breastfeeding, even for a short period of time, may counterbalance the risk of obesity even in genetically predisposed adolescents by inducing epigenetic modifications^5^. Other studies have shown that the docosahexaenoic acid (DHA) content of human milk is associated with better cognitive development of infants and children^6-7^. DHA is an important component of the retinal photoreceptors and of the brain cell membranes and hence represents a significant factor for the visual and cognitive development of the individual^8^. Since the DHA status in infancy may fundamentally impact on long-term health, the European Food Safety Authority has acknowledged this epigenetic effect and has recommended an intake of 100 mg/day of DHA for infants and young children up to the age of 24 months old^9^.

There is a growing body of evidence in the literature supporting that maternal diet can affect the composition of human milk and specifically its fat, fatty acid and vitamin content^10^. Fat has been proven to be a critical component of breast milk by providing energy and important nutrients that are fundamental to the development of the central nervous system and cannot be synthesized de novo by the infant^11^. A meta-analysis of 65 studies worldwide found that DHA is the component of human milk that varies greatly among populations^12^. The meta-analysis reported that the highest DHA concentrations were primarily in coastal populations and were associated with high marine food consumption.

There are reports that the Mediterranean diet has a positive effect on the fat and fatty acid composition of human milk. A study from Greece has shown that even for a long period of lactation the DHA content of human milk remained higher than in other countries^11^. This study showed that the specific characteristics of the Mediterranean diet, which involves an increased intake of fat and mainly mono-unsaturated fat intake and carbohydrate intake, led to a breast milk content that was higher in DHA, arachidonic acid and total poly-unsaturated fatty acids compared to mother’s milk in other European countries. The authors suggested that Greek women while in pregnancy or lactation choose a diet with more natural and organic products, such as wild greens and herbs, open sea fish, and free-range chickens, all of which contribute to an increased ω-3 fatty acid intake.

Moreover, a recent systematic review of 59 observational and 43 interventional studies reported that the maternal dietary intake of fatty acids, fat soluble vitamins, vitamin B1 and vitamin C, was related to their content in breast milk composition^13^. It has also been reported that the provision of vitamin A to the newborn through breast milk is mostly affected by the maternal dietary intake, even more than the transfer of this vitamin through the placenta to the fetus^14^. Another study has shown that the breast milk’s content in vitamin E positively correlates with the maternal dietary fat intake^15^.

In view of the epigenetic effects of human milk and the potential to modify the nutrient content of human milk through dietary modifications, it is imperative that midwives provide appropriate nutritional advice to women, as they are the frontline professionals of antenatal and postnatal care provision to mothers. There are reports in the literature concerning the efficacy of interventions by midwives on dietary habits during pregnancy^16^. It should be highlighted that there is no need for restrictive diets; an increase of maternal energy intake of approximately 500 kcal per day is more than enough to cover the extra energy expenditure of lactation^17^. A healthy diet should be promoted that will ensure the high intake of vitamins, minerals and fiber that are important for the lactating mother. Such a diet could be the Mediterranean diet, which involves increased vegetable oil consumption, mainly olive oil, increased carbohydrates, a variety of fruit and vegetables, and more natural foods as possible^11,17-18^.

Moreover, midwives need to give advice on the necessity of lactating mothers ensuring a daily intake of at least 200 mg DHA^18^. This can be achieved either through maternal dietary intake from an appropriate diet source or through supplements. The Food and Drug Administration in the United States suggests that lactating mothers should consume certain types of fish with naturally occurring ω-3 fatty acids and high levels of DHA (i.e. herring, salmon, mackerel, sardines, anchovies)^19^. The American College of Obstetricians and Gynecologists recommends that, given the concerns for mercury toxicity with overconsumption of certain fish, in order for women to meet their dietary intake recommendations they will need to consume ω-3 fatty acids from three different categories of foods that include vegetable oils (i.e. flaxseed oil, canola oil, soybean oil), two servings of seafood per week, or take ω-3 fatty acid supplements containing DHA^20^. Given that several studies have reported that it is difficult for a lactating woman to reach the advised DHA intake only through her diet, it is still not clear what the ideal supplementation dose of DHA in lactation should be^20^.

The literature shows that the midwives’ consultation during pregnancy on issues of life style and maternal diet is effective in promoting healthier habitual behaviors^16^. Furthermore, midwives are the key healthcare professionals to promote breastfeeding and provide guidance and support to lactating mothers postpartum. In the context of providing holistic care to their clientele, midwives need to provide adequate and up to date nutritional advice during pregnancy and lactation, in order to promote maternal and neonatal health as well as quality breast milk content.

## CONFLICTS OF INTEREST

Authors have completed and submitted the ICMJE Form for Disclosure of Potential Conflicts of Interest and none was reported.
